# Dosimetric Characterization Using Quantitative Magnetic Resonance Imaging of PAGAT and Gd-PAGAT Polymer Gel Dosimeters at High Gd Concentration

**DOI:** 10.3390/gels12080745

**Published:** 2026-08-20

**Authors:** Melani Fuentealba, Gerardo Belmar, Mauricio Santibáñez

**Affiliations:** 1 Departamento de Cs. Físicas, Universidad de La Frontera, Temuco 4811230, Chile; 2 Laboratorio de Metrología de Radiaciones Ionizantes, Universidad de La Frontera, Temuco 4811230, Chile; 3 Departamento de Tecnología Médica, Universidad de Concepción, Concepción 4070386, Chile

**Keywords:** gel dosimetry, PAGAT, Gd-PAGAT, quantitative magnetic resonance, R_2_ mapping, spectrophotometry, dose enhancement, gadolinium

## Abstract

Gadolinium-doped polymer gel dosimeters enable experimental evaluation of dose enhancement in low-energy radiotherapy, although the high Gd concentrations required substantially alter their magnetic relaxation properties, making the optimal Magnetic Resonance Imaging (MRI) readout uncertain. This study characterized the dosimetric response of PAGAT and Gd-PAGAT (9 mg/mL Gd) irradiated with a 150 kVp X-ray beam from 1 to 10 Gy using quantitative 1.5 T MRI. Quantitative R_1_ and R_2_ maps were obtained from variable flip-angle spoiled gradient echo (SPGR) and multi-echo spin echo (MSE) sequences, respectively, and validated by UV-Vis spectrophotometry. R_1_ showed no significant dose dependence in Gd-PAGAT because of severe T_1_ shortening. In contrast, R_2_ exhibited a robust response, with second-order polynomial fits (R^2^ = 0.9984 for PAGAT and 0.9969 for Gd-PAGAT) and linear behavior between 1 and 7 Gy. The dose sensitivity of Gd-PAGAT was 2.84 ± 0.19 times higher than that of PAGAT (0.5328 vs. 0.1949 s^−1^·Gy^−1^). Spectrophotometry confirmed the PAGAT calibration but showed markedly lower sensitivity for Gd-PAGAT, particularly at low doses. These findings demonstrate that R_2_ mapping overcomes the limitations imposed by extreme T_1_ shortening, extending the applicability of Gd-PAGAT dosimeters for three-dimensional dose enhancement studies in low-energy radiotherapy.

## 1. Introduction

Three-dimensional polymer gel dosimetry is the only experimental strategy capable of recording the complete volumetric absorbed dose distribution in clinically realistic geometries, offering submillimeter spatial resolution and radiological soft-tissue equivalence [1]. This capability is critical in scenarios characterized by steep dose gradients or where the three-dimensional symmetry of the radiation field cannot be assumed a priori. Such scenarios include stereotactic radiosurgery, electronic brachytherapy (eBT), and intraoperative radiotherapy (IORT) [2]. Among the available normoxic formulations, PAGAT (PolyAcrylamide Gelatin with THPC) has established itself as one of the most widely studied options due to its favorable compromise between limited toxicity, post-irradiation stability, and a linear response within the therapeutic range of 2 to 10 Gy [3].

Polymer gel dosimeters can be read out by multiple imaging modalities, including optical spectrophotometry, X-ray computed tomography, and quantitative magnetic resonance imaging (MRI), the latter being the most widely used [4]. MRI yields three-dimensional, voxel-by-voxel dose maps by quantitatively fitting the longitudinal (R_1_) and transverse (R_2_) proton relaxation rates of water, where R_1_ = 1/T_1_ and R_2_ = 1/T_2_. In undoped PAGAT, radiation-induced polymerization restricts the rotational mobility of water molecules in the vicinity of the newly formed polymer network, which selectively increases R_2_ while the R_1_ remains practically insensitive to dose within the therapeutic range. Reported dose–R_2_ sensitivities for gel dosimeters at 1.5 T are in the order of 0.15–0.30 s^−1^·Gy^−1^ [3,5], establishing the multi-echo spin-echo (MSE) sequence as the natural choice for the quantitative readout of these dosimeters, a principle recently extended to real-time 4D dosimetry on MR-Linac systems [6].

The consistency between quantitative MRI and UV-Vis spectrophotometry on identical samples has been established for several polymer formulations. Both modalities respond to radiation-induced polymerization with quantifiable, dose-dependent sensitivities [7,8], and the R_2_-versus-dose fit can yield a slightly better coefficient of determination and a lower detection limit than optical absorbance [9]. This consolidates quantitative MRI as the primary reference for gel dosimetry and positions UV-Vis spectrophotometry as a suitable independent method for cross-corroborating dose–response calibrations, particularly where optical behavior may deviate from relaxation behavior due to polymer matrix or doping effects.

An increasingly clinically relevant line of research explores the infusion of polymer gels with high-atomic-number (Z) elements, including gadolinium (Z = 64), with the aim of experimentally quantifying the physical dose enhancement produced in the vicinity of tissues selectively doped with high-Z agents and irradiated with low-energy beams [10,11]. Gadolinium is particularly attractive in this context because it is available in pharmaceutical formulations approved for clinical use as MRI contrast agents, facilitating its availability and clinical applicability. Furthermore, its K-absorption edge is located at 50.2 keV, which is within the optimal energy range for eBT, IORT, and kilovoltage modalities. Additionally, the L- and M-edges at lower energies contribute to dose enhancement even below the K-edge [12]. The gadolinium concentrations required to produce a measurable dosimetric increase typically lie in the range of several mg/mL [13]. This is two to three orders of magnitude above the characteristic tissue regime in clinical MRI, where contrast agent concentrations are maintained within the range of ~0.1 to 1 mM (~0.016 to 0.16 mg/mL) [14].

Over the past decade, interest in Gd-doped dosimetric gels has grown, characterizing their response across a wide range of concentrations (up to 40 mg/mL) under kilovoltage and megavolt X-ray beams [13,15,16,17,18]. The same pattern extends to gels loaded with metallic nanoparticles or high-Z oxides (Au, Bi, ZnO, Pt) into MAGAT or PAGAT matrices [19,20]; in the vast majority of these works the readout is optical (UV-Vis spectrophotometry or optical CT), with MRI absent or reduced to a secondary reference.

The incorporation of Gd profoundly modifies the nuclear relaxation of the medium alongside physical dose enhancement. As a highly efficient paramagnetic agent (seven unpaired 4f electrons), Gd^3+^ shortens both relaxation rates, but has a dominant effect on T_1_ within the linear relaxivity regime of its clinical use [14]. This makes a specific behavior plausible for Gd-PAGAT: if the Gd dictates the relaxation mechanism, the dosimetric modulation of the polymer network could transfer from the R_2_ channel, dominant in pure PAGAT, to the R_1_ channel, reversing the logic of the optimal readout sequence. In the same direction, at the several mg/mL required for physical enhancement, the Gd contribution globally shortens T_2_, which could compromise the signal-to-noise ratio (SNR) of multi-echo T_2_-weighted images and reduce the viability of the spin-echo sequence as a quantitative method.

This expectation does not necessarily hold at high concentration, where the MRI signal becomes harder to predict. The signal-versus-concentration curve of T_1_-weighted spoiled gradient echo (SPGR) sequences is markedly non-linear. As the Gd increases, T_1_ shortens to a few milliseconds, longitudinal magnetization recovers completely between successive TRs, and the signal saturates [21,22]. For common Gd chelates, the signal peaks around 2.5 mmol/L between 1.5 and 7 T [23]. At the tens of mmol/L required for dosimetric enhancement, the system operates far beyond this peak, a regime where the quantitative SPGR readouts may become useless. The shift in the sensitive channel toward T_1_ and its masking by T_1_ saturation thus remain experimentally unresolved for Gd-doped gels in the dosimetric range.

The closest precedents do not resolve these competing predictions. The few works that characterize Gd-doped gels by MRI operate at contrast-range concentrations, orders of magnitude below the dosimetric level: De Deene and Mason (2023) [6] optimized MRI sequences on a gadobutrol-doped MAGAT dosimeter for real-time 4D dosimetry, yet at concentrations tuned for acquisition speed rather than for dose-enhancement sensitivity [6]. An experimental gap therefore persists for the quantitative MRI characterization of a Gd-doped gel at concentrations of several mg/mL.

This work directly addresses this experimental gap. We report the dosimetric characterization at 1.5 T via quantitative MRI of PAGAT and Gd-PAGAT gels at a gadolinium concentration of 9 mg/mL, irradiated with a 150 kVp X-ray beam in the range of 1 to 10 Gy. The responses in the R_1_ and R_2_ channels are comparatively evaluated using MSE sequences for T_2_ and SPGR sequences at multiple flip angles for T_1_. The MRI characterization is complemented by UV-Vis spectrophotometry readouts on the same irradiated samples, following the methodological approach established by prior studies [7,8,9], as an independent cross-corroboration of the dose–response calibration. The objectives are to empirically identify the dose-sensitive relaxation channel in the high-concentration Gd regime, quantify the sensitivity amplification relative to pure PAGAT, and establish an experimental foundation for designing optimized MRI protocols for gel dosimetry with dose enhancement in electronic brachytherapy, IORT, and other low-energy modalities.

## 2. Results and Discussion

### 2.1. Conventional PAGAT Response in T_2_

**Figure 1:**
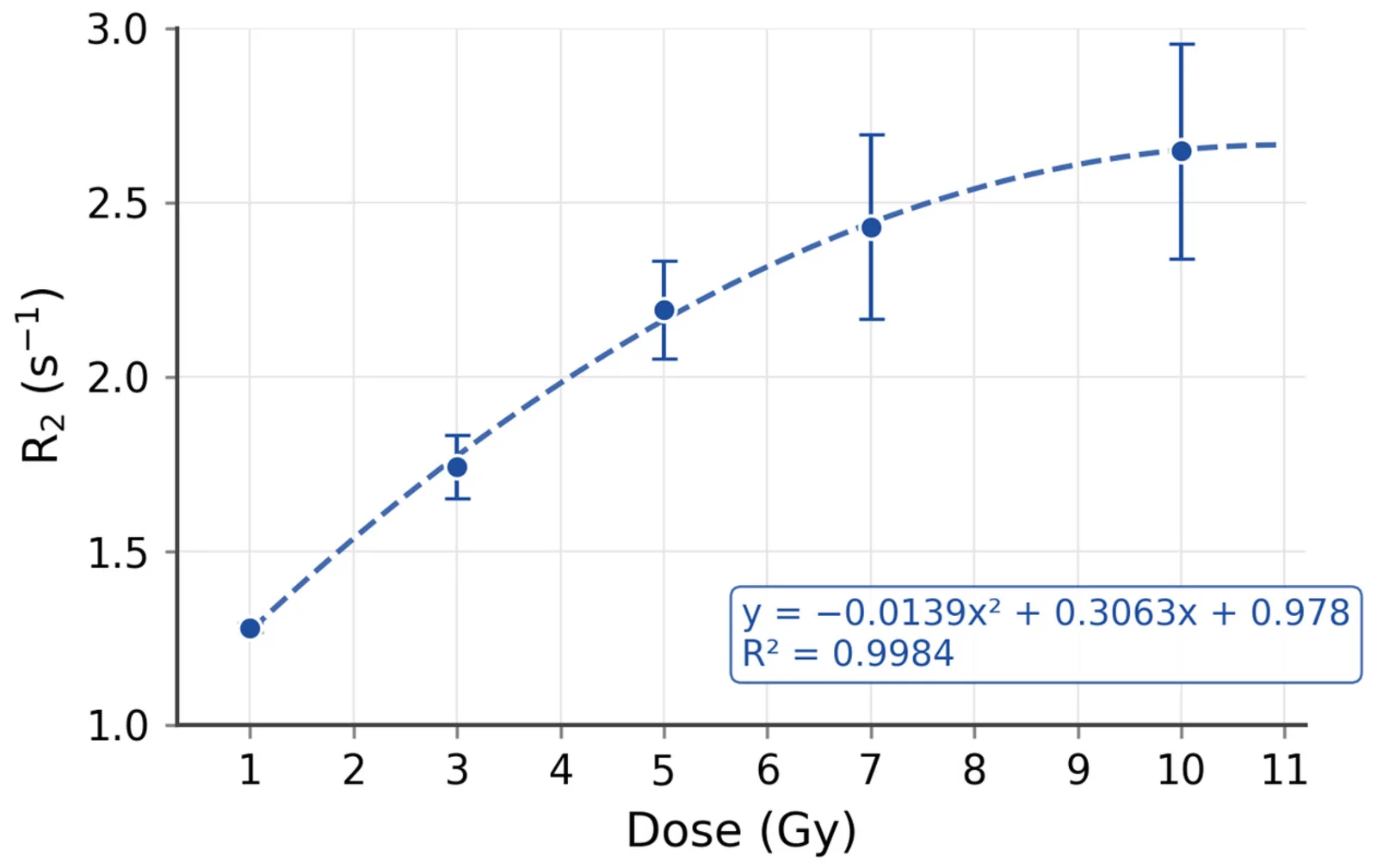
Dose–response curve of PAGAT processed via MRI, with expanded uncertainty at a coverage factor of k = 2.

The conventional PAGAT dosimeter exhibited a monotonically increasing correlation between the absorbed dose and the R_2_ values derived from the MSE sequence (Figure 1). This behavior is consistent with the established mechanism for undoped polyacrylamide gels, in which radiation-induced polymerization restricts the rotational mobility of water molecules and selectively manifests as an increase in the transverse relaxation rate [6]. In contrast, the SPGR-derived R_1_ channel yielded no detectable dosimetric correlation within the evaluated range, confirming that T_2_-weighting constitutes the only robust quantitative pathway for the MRI readout of PAGAT in the absence of paramagnetic doping.

The average R_2_ values obtained per ROI for each dose level are presented in Table 1. The second-degree polynomial fit over the entire 1 to 10 Gy range described the dosimetric response according to Equation (1),

(1) *R*_2_(*D*) = −0.0139 · *D*^2^ + 0.3063 · *D* + 0.978 (*s*^−1^),

with a coefficient of determination of R^2^ = 0.9984. When restricting the fitting domain to the 1–7 Gy sub-range, a linear regression yielded R^2^ = 0.9815 with a slope of 0.1949 s^−1^·Gy^−1^ (95% CI [0.114, 0.276]). Starting from 10 Gy, the onset of the saturation regime becomes apparent, which is expected for PAGAT formulations at this monomeric concentration and is consistently reported in the literature [24]. The expanded uncertainty (k = 2) shown as error bars derives from the standard deviation of R_2_ among the ROIs within each vial; it therefore quantifies the spatial variability of the dosimeter response (polymerization heterogeneity) rather than the acquisition noise of the scanner. The larger dispersion at higher doses is consistent with this origin: the spatial standard deviation of R_2_ scales with the degree of polymerization, because the size and density of the polymer microgels increase with dose, sharpening the local conversion gradients [25,26]. At low doses the incipient polymer network is spatially more homogeneous, yielding the smaller inter-ROI dispersion observed.

### 2.2. Gd-PAGAT Response at 9 mg/mL: T_1_ vs. T_2_

Contrary to the expectation based on the paramagnetic nature of the Gd^3+^ ion, which traditionally drives T_1_ shortening as the dominant contrast mechanism, the T_1_-weighted images acquired with the SPGR sequence did not allow for the identification of any detectable correlation between the absorbed dose and the R_1_ map. Within experimental dispersion, the pixel intensity at a fixed TR and flip angles (α) of 15° and 30° showed no monotonic trend with dose.

**Figure 2:**
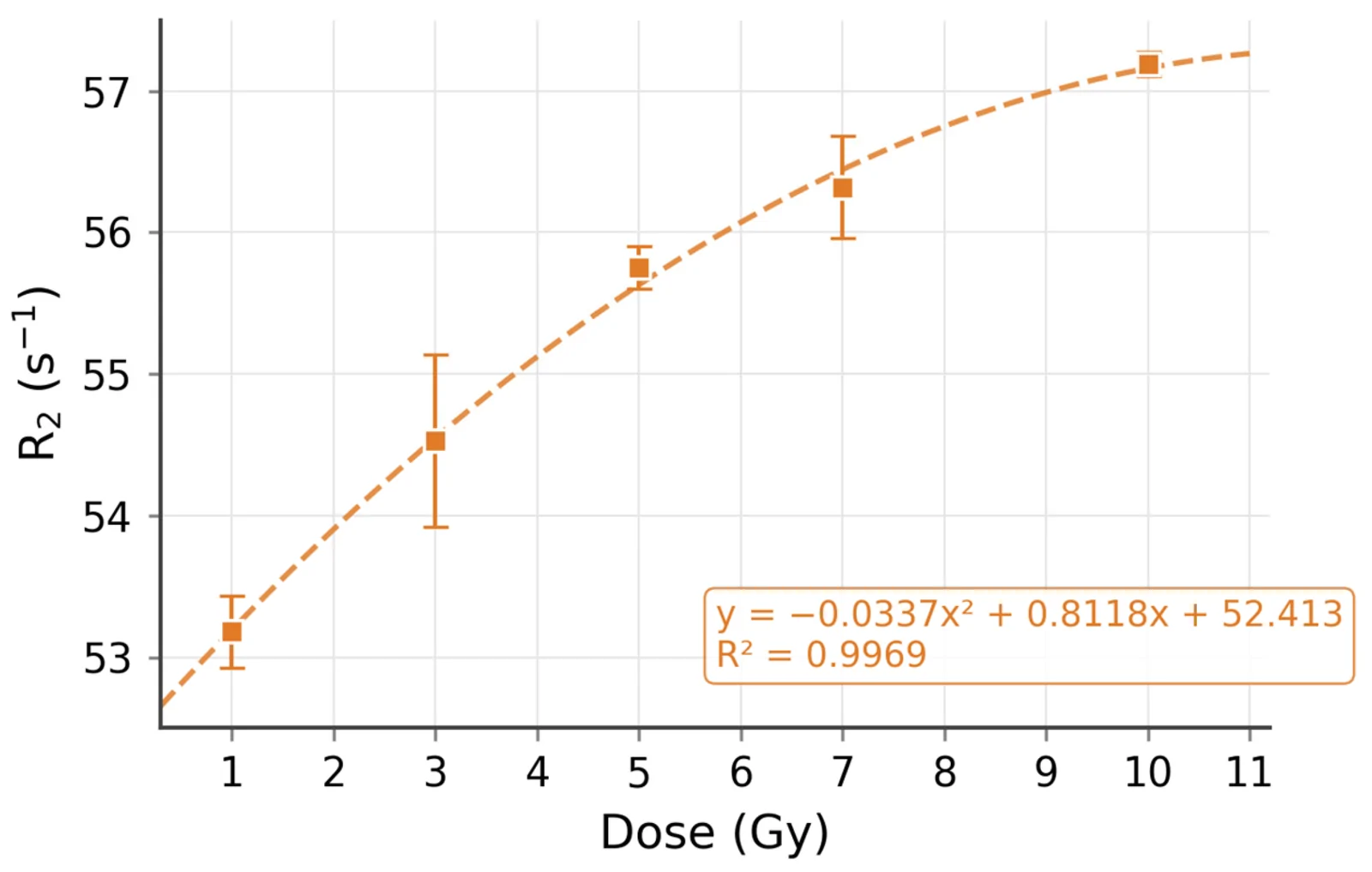
Dose–response curve of Gd-PAGAT processed via MRI, with expanded uncertainty at a coverage factor of k = 2.

In contrast, the MSE T_2_-weighted images did allow for the extraction of an R_2_ map with a robust correlation to dose (Figure 2). The second-degree polynomial fit for the Gd-PAGAT at 9 mg/mL in the 0–10 Gy range was:

(2) *R*_2_(*D*) = −0.0337 · *D*^2^ + 0.8118 · *D* + 52.413 (*s*^−1^)

This yielded R^2^ = 0.9969. When restricting the fitting domain to the 1–7 Gy sub-range, a linear regression achieved R^2^ = 0.9718 with a slope of 0.5328 s^−1^·Gy^−1^ (95% CI [0.257, 0.809]).

The absolute values of the R_2_ map of Gd-PAGAT are substantially higher than those of PAGAT at the same dose (Table 1), reflecting the paramagnetic contribution of unirradiated Gd to the baseline value of R_2_(0) = 52.41 s^−1^, which exceeds the baseline value of PAGAT (R_2_(0) = 0.993 s^−1^) by approximately 53-fold. The correlation, although robust, proved to be more sensitive to the size and location of the ROI than that of conventional PAGAT. The average deviation obtained within the evaluated ROI of PAGAT was 0.042 s^−1^·Gy^−1^, whereas for Gd-PAGAT, the average deviation of the values within the same ROI increased to 0.68 s^−1^·Gy^−1^. Consequently, an analysis of smaller ROIs centered on the region of maximum polymerization is required.

### 2.3. Signal Amplification and Sensitivity

The average amplification factor over the evaluated dose points (1–10 Gy) is 28.6×, exhibiting a decreasing behavior with dose (41.5× at 1 Gy and 21.6× at 10 Gy), reflecting the earlier saturation of Gd-PAGAT. However, these values include the high baseline value of Gd-PAGAT, which is independent of the dose (0 Gy = 52.4 s^−1^).

**Figure 3:**
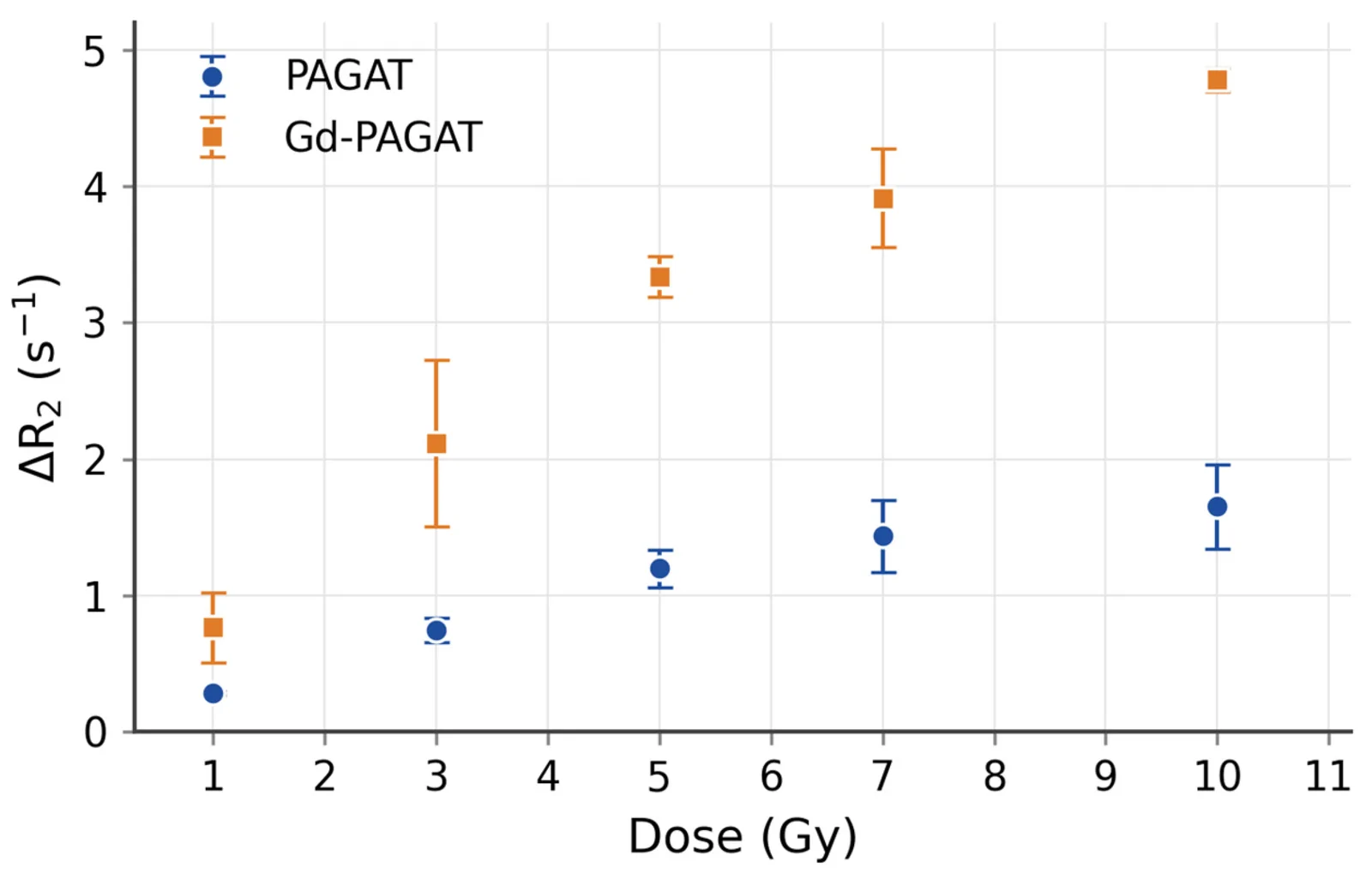
Comparative dose–response curve of PAGAT (blue) and Gd-PAGAT (orange) showing the effective increase in R_2_ attributable to the dose.

The difference between both dosimeters attributable to the dose was defined by subtracting the baseline contribution of the unirradiated Gd-PAGAT, thereby isolating the R_2_ response associated with the radiation-induced polymerization process (as shown in Table 1). Figure 3 presents the comparison of ΔR_2_ versus dose for both formulations, showing a largely constant Gd-PAGAT/PAGAT ratio of 2.84 ± 0.19 over the 1–10 Gy range, where the reported dispersion corresponds to the standard deviation among the five evaluated dose points. This value is consistent with the ratio of the slopes obtained from the linear fits (0.5328 s^−1^·Gy^−1^ for Gd-PAGAT versus 0.1949 s^−1^·Gy^−1^ for PAGAT, a 2.73× ratio), confirming that the amplification operates homogeneously across the entire dose–response curve.

### 2.4. Corroboration by UV-Vis Spectrophotometry

**Figure 4:**
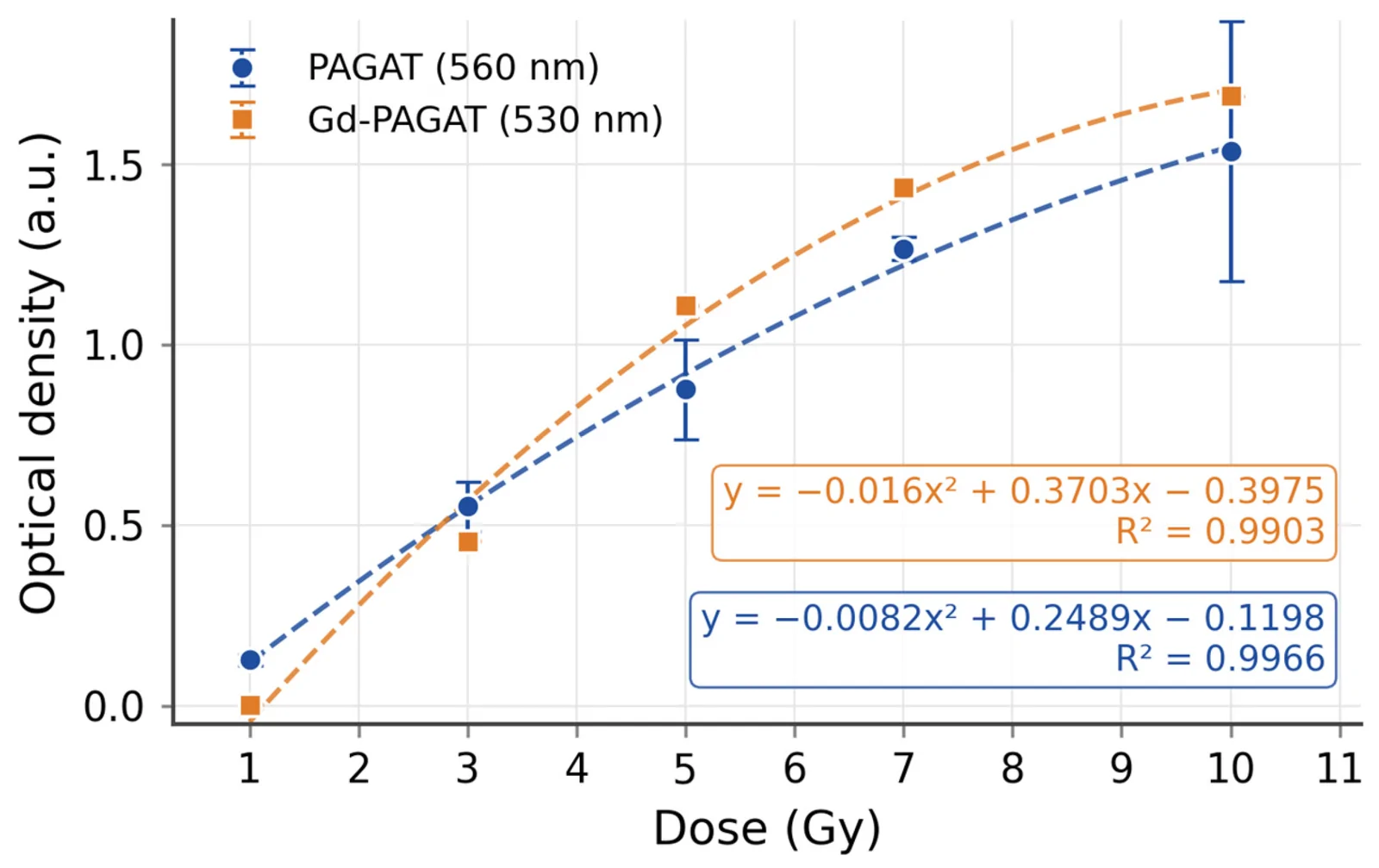
Comparative dose–response curve of PAGAT (blue) and Gd-PAGAT (orange) processed via optic spectrophotometry.

The spectrophotometric readings at 560 nm (PAGAT) and 530 nm (Gd-PAGAT) performed on the same irradiated samples are presented in Table 2. The selection of wavelengths was based on the maximum optical sensitivity observed in preliminary spectral scans. Both dosimeters exhibited a monotonic increase in OD with dose (Figure 4), in qualitative agreement with the response observed via MRI.

The linear fit in the 1–7 Gy range yields a slope of 0.187 and an R^2^ of 0.9966 for PAGAT, whereas for the 1–10 Gy dose range, a second-degree polynomial fit yields

(3) *OD*(*D*) = −0.0082 · *D*^2^ + 0.2489 · *D* − 0.1198

with an R^2^ of 0.9966. For Gd-PAGAT, within the 1–7 Gy dose range, the linear fit gives a slope of 0.2473 Gy^−1^, with an R^2^ of 0.9857. This improves noticeably when transitioning to a second-degree polynomial fit over the 1–10 Gy range,

(4) *OD*(*D*) = −0.016 · *D*^2^ + 0.3703 · *D* − 0.3975

with an R^2^ of 0.9903. The lower quality of the linear fit for Gd-PAGAT is dominated by the low optical response behavior observed at 1 Gy (OD = 0.0045), an aspect that is discussed below.

Comparing the slopes obtained from MRI and spectrophotometry reveals a 4.05% difference between the two processing methods for conventional PAGAT, indicating that spectrophotometry is a reliable secondary corroboration method. In contrast, for Gd-PAGAT, a 53.58% difference is observed between their slopes, indicating a loss of sensitivity in spectrophotometry at both low doses (1 Gy) and doses above 7 Gy.

The divergence for Gd-PAGAT is a consequence of the two observables probing different physical manifestations of the same polymerization process: R_2_ responds to the restriction of water mobility at the molecular scale, whereas optical density depends on scattering by polymer–Gd aggregates that require a minimum size to scatter appreciably at 530 nm. This produces two regimes for Gd-PAGAT: at 1 Gy the optical response is almost null (OD = 0.0045 ± 0.001) because the aggregates have not yet reached the scattering size, while R_2_ already shows a robust signal; above 7 Gy the optical response saturates earlier than R_2_. The divergence therefore concentrates at both ends of the range, and MRI remains the reference readout for Gd-PAGAT, with spectrophotometry at 530 nm serving as a qualitative complement rather than a quantitative corroboration.

### 2.5. Discussion

Under the evaluated conditions, the SPGR sequence gave no dose–R_1_ correlation for Gd-PAGAT at 9 mg/mL, while the MSE sequence gave a robust dose–R_2_ correlation. These findings discriminate between two a priori hypotheses: that the paramagnetic Gd^3+^ ion would shift the dose-sensitive channel from R_2_ to R_1_, and that the marked T_1_ shortening produced by Gd^3+^ at several mg/mL would saturate the SPGR signal. The results support the latter: the markedly shortened baseline T_1_ drives the SPGR signal into a saturation regime, and since radiation-induced polymerization barely modifies R_1_ even in undoped PAGAT, the dose-induced change in R_1_ would be too small to resolve against the Gd-dominated baseline. The absence of a dose–R_1_ correlation would thus reflect the limits of this single weighting scheme; SPGR-based T_1_ mapping is therefore not a direct dosimetric readout for high-Gd gels, and the readout must rely on T_2_.

The T_2_ readout, although robust, shows a larger intra-ROI dispersion for Gd-PAGAT than for PAGAT, with two causes. The first is instrumental: both formulations were acquired with a common echo train despite their very different T_2_ values, so the late echoes of Gd-PAGAT fall near the noise floor; this is a limitation of the protocol, not of the dosimeter, and an echo train adapted to its shorter T_2_ would restore precision [27,28]. The second is intrinsic: because R_2_ is dominated by Gd, small inhomogeneities in Gd concentration produce R_2_ variations proportional to the elevated baseline, a term that only improved gel homogeneity can mitigate.

The dose-attributable metric is the dose–response slope, 2.84 ± 0.19 times higher for Gd-PAGAT than for PAGAT (Table 3); the larger absolute R_2_ ratio (28.6×) merely reflects the elevated baseline. This amplification must be read in light of the device’s purpose: an integrated dosimeter–phantom in which gel and a high-Z agent form a single, initially tissue-equivalent volume meant to register the extra dose the agent deposits. The higher R_2_ would therefore reflect primarily this physical dose enhancement rather than a chemical effect, as previously characterized for Gd-PAGAT by Monte Carlo simulation [12].

Spectrophotometry on the same samples agreed with the MRI calibration for PAGAT (slopes within 4%) but not for Gd-PAGAT (53.58% difference). Both R_2_ and optical density stem from the same polymerization but probe different manifestations of it: molecular water mobility versus scattering by polymer–Gd aggregates that require a minimum size to scatter at 530 nm. This would explain the near-null optical response of Gd-PAGAT at 1 Gy against its clear R_2_ signal, giving the optical readout a lower sensitivity limit at low dose. Rather than mutual confirmation, this indicates that for Gd-PAGAT at a very low dose MRI is preferable, with spectrophotometry at 530 nm serving as a qualitative complement.

Two limitations frame these results. Only one T_1_-weighting sequence and a single Gd concentration were evaluated, so the response should not be extrapolated to the whole high-concentration regime; 9 mg/mL was chosen as it lies within the range expected to yield a measurable enhancement while remaining physiologically achievable, yet is already high relative to previously reported Gd-doped gel dosimeters. As a proof-of-concept, no comparison with an established dose-enhancement dosimeter was possible because none yet exists, which is the gap these developments aim to fill; validation with complex three-dimensional dose distributions is the necessary next step.

## 3. Conclusions

Quantitative 1.5 T MRI characterization of PAGAT and Gd-PAGAT dosimeters at 9 mg/mL, about two orders of magnitude above the concentration used for MRI contrast agents, shows that, under the evaluated conditions (a single SPGR scheme at fixed TR and two flip angles), T_1_-weighting is not a viable pathway for extracting dosimetric information; other sequences, field strengths or optimized T_1_ protocols could modify this behavior. In contrast, T_2_-weighting via MSE delivers a robust and reproducible dose–R_2_ response, with a dose sensitivity 2.84 ± 0.19 times higher for Gd-PAGAT than for PAGAT over 1–10 Gy. UV-Vis spectrophotometry on the same samples agreed with the MRI calibration for PAGAT (within 4%) but not for Gd-PAGAT, where the two readouts diverged; for this formulation, spectrophotometry at 530 nm provides valuable complementary optical data, while MRI serves as the primary readout for full-range quantitative evaluation. These results redefine the operational space of Gd-PAGAT gels for 3D dose-enhancement dosimetry and show that high Gd concentrations within the dosimetrically useful range can be explored without compromising the MRI readout, provided the sequence is shifted toward the T_2_ channel.

## 4. Materials and Methods

### 4.1. Dosimeter Preparation

PAGAT dosimeters were prepared following the normoxic protocol described by Venning et al. (2004) [29], with a nominal composition by weight (%*w*/*w*) of 5.0% gelatin, 88.7% Milli-Q water (Merck Millipore, Darmstadt, Germany), 3.0% N,N’-methylenebisacrylamide (BIS), 3.0% acrylamide, and 10 mM of THPC as an oxygen scavenger. The preparation was carried out in a thermostatic bath at temperatures between 37 and 50 °C depending on the stage, under magnetic stirring and inside a fume hood; THPC was incorporated in the final stage two minutes before packaging.

For the Gd-PAGAT dosimeters, the stoichiometric amount of the gadolinium-based contrast agent was introduced alongside the THPC in the final stage, proportionally adjusting the volume of water added in the last step to achieve a nominal concentration of 9 mg/mL of Gd in the final mixture. This strategy prevents early reactions between Gd and the monomers and maintains the proportions of the remaining components.

The gadolinium contrast agent used for infusing the PAGAT dosimeters was Omniscan (GE HealthCare, Chicago, IL, USA), a linear non-ionic agent whose active ingredient is gadodiamide and features Gd-DTPA (gadolinium dihydroxypropylenediamine pentacetate acid) as the chelating agent.

The dosimeters were packaged in transparent polystyrene spectrophotometry vials with internal dimensions of 4.5 × 1.0 × 1.0 cm^3^ and stored refrigerated at 4 °C for at least 24 h prior to irradiation to ensure complete gelatinization. Two vials were prepared for each combination of dosimeter type (PAGAT, Gd-PAGAT) and nominal dose level.

### 4.2. Irradiation System

A low- and medium-energy X-ray calibration beamline was utilized, equipped with a Teledyne model CP160D industrial X-ray tube (Teledyne ICM, Dison, Belgium). This generator operates with tunable potentials from 10 to 160 kV in 1 kV intervals, tube currents of 1–10 mA, and a maximum power of 900 W. The system features a tungsten anode and a 0.8 mm beryllium emission exit window. Positioned in front of the beam exit is a radiation beam-shaping system that houses a primary collimator, whose function is to limit the output beam from the X-ray tube to the active area of a model 96,035 monitor chamber (Keithley Instruments, Solon, OH, USA), used to monitor and correct fluctuations across the different irradiations. Between the primary collimator and the monitor chamber, a slot is available for inserting inherent Al and Cu filters to establish the desired beam quality during irradiation. Finally, a removable secondary collimator is located on the external part of the structure to establish the field size at the sample to be irradiated, for ranges up to 1.5 × 1.5 cm^2^.

The configuration employed during dosimeter irradiation consisted of a UW-150M beam quality (150 kVp and an HVL of 10.3 mm Al), a 5 × 5 cm^2^ field, and an SSD of 25 cm. The vials were placed at a depth of 1.5 cm (2.0 cm measured relative to their center) inside a 20 × 20 × 12 cm^3^ Virtual Water phantom to provide the required lateral scatter and backscatter conditions, generating a dose rate of 0.925 ± 0.019 at the center of the dosimeters. To ensure a homogeneous dose distribution throughout the volume of the dosimeter, the irradiation was fractionated into four orthogonal fields, rotating the vials 90° between exposures. The nominal dose points used ranged from 1 to 10 Gy, in addition to an unirradiated control sample.

Reference dosimetry to determine the dose rate delivered to the dosimeters was performed following the TRS-398 rev1 Code of Practice [30], using a Farmer-type ionization chamber (model A12) from Standard Imaging (Standard Imaging, Inc., Middleton, WI, USA) and a max4000 electrometer from the same brand, calibrated in terms of air kerma in air for UW-150M beam quality. For medium-energy X-ray beams (tube potential > 80 kVp and HVL > 2 mm Al), this protocol prescribes reference dosimetry in a water phantom at a reference depth of 2 cm (2 g/cm^2^), with the phantom extending at least 5 cm beyond the field in the directions perpendicular to the beam to provide full lateral scatter and 10 cm beyond the measurement depth to provide full backscatter. The absorbed dose to water is obtained from the air-kerma calibration coefficient through the factor [μ_en_/ρ]_w,air_, the spectrum-averaged water-to-air ratio of mass energy-absorption coefficients at 2 cm depth, tabulated in the protocol as a function of tube potential, HVL, SSD and field size; the chamber factor p_ch,Q_, which accounts for the change in chamber response between measurements at 2 cm depth in water and in air, tabulated for specific chambers approved for reference dosimetry (here the Exradin A12); and the beam-quality conversion factor k_Q,Q0_ between the calibration quality of the air-kerma coefficient and the irradiation quality, which equals unity here because the calibration quality (UW-150M) was reproduced during irradiation.

### 4.3. Magnetic Resonance Acquisition

**Figure 5:**
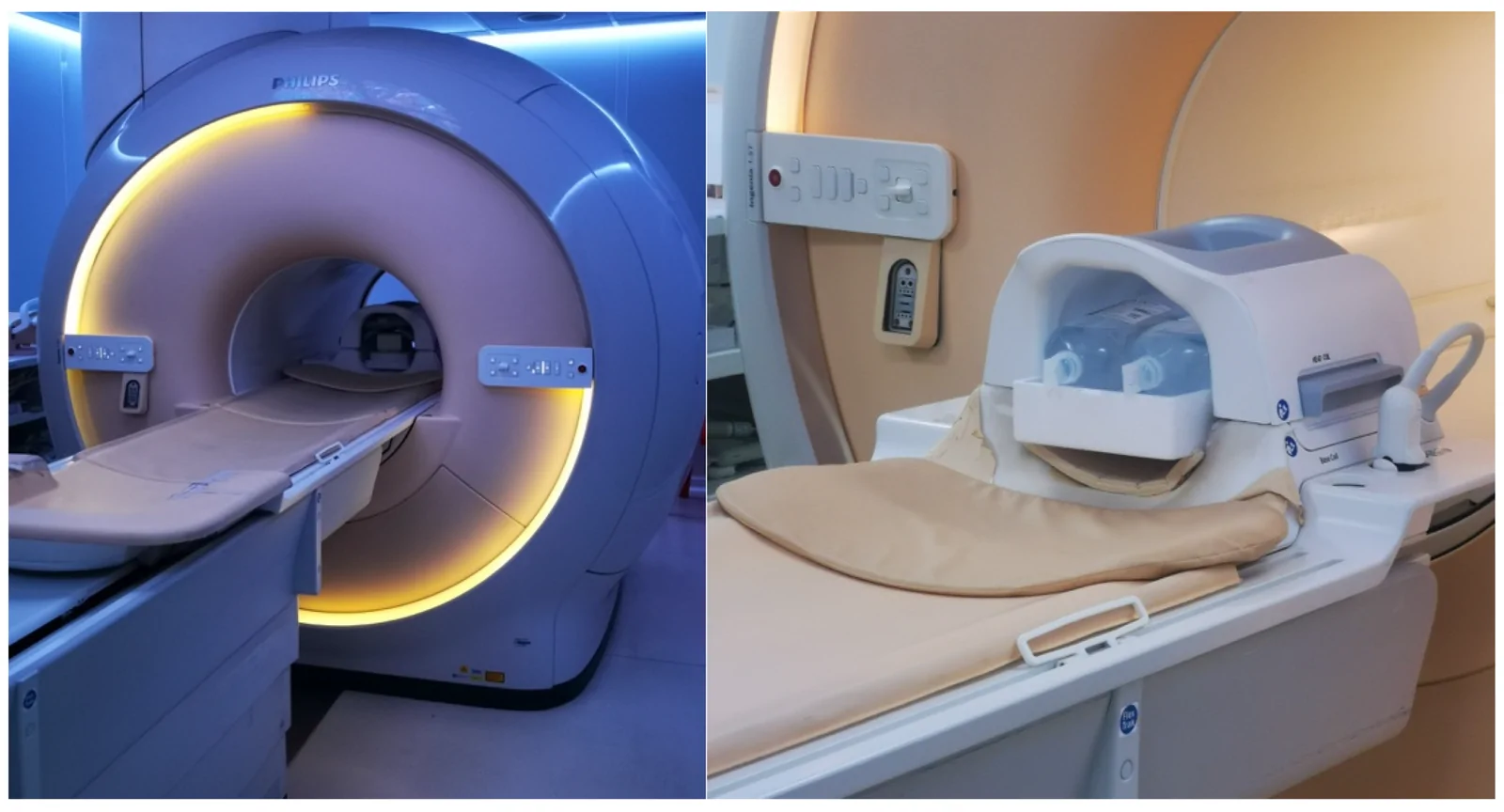
Philips Ingenia scanner (**left**) with PAGAT and Gd-PAGAT samples. To increase the signal, 1000 mL of saline solution was added (**right**).

Images were acquired on a 1.5 T Philips Ingenia scanner (Philips Healthcare, Best, The Netherlands), used for both T_1_-weighted and T_2_-weighted sequences (Figure 5). All vials (PAGAT and Gd-PAGAT across all doses and controls) were scanned in a single session inside a head coil, minimizing inter-session variability due to ambient temperature and equipment drift. An MSE sequence was used for T_2_-weighting, and an SPGR sequence was used for T_1_-weighting.

The acquisition parameters of the sequences used for R_1_ and R_2_ quantification are summarized in Table 4. The differences in spatial resolution, slice thickness, and acquisition matrix between the MSE and variable flip angle SPGR sequences are inherent to the design of each technique and their specific constraints regarding signal-to-noise ratio, total acquisition time, and k-space sampling efficiency. Full harmonization of these parameters between sequences is not feasible without compromising the quantitative quality of at least one of the modalities. Instead, the rigid registration between image spaces, described in Section 4.4, allowed the integration of the parametric maps into a common coordinate system while maintaining the quantitative fidelity of each sequence in its optimal regime.

Images were acquired in the three orthogonal planes with an average voxel size of 0.90 × 1.14 × 1.00 mm^3^ and a slice spacing of 3 mm. The field of view (FOV) was adjusted to the constraints of each combination.

### 4.4. Processing and Fitting of Relaxation Maps

Image processing was performed using the open-source software 3D Slicer (version 5.8.1) integrated with the Python (version 3.11) routines developed in this work. Since the T_1_-weighted and T_2_-weighted series were acquired with different spatial resolutions (Table 4), the T_2_ images were co-registered to the space of the T_1_ images via a rigid registration implemented with the Transforms module of 3D Slicer, adopting the latter as the geometric reference due to its higher resolution. On the resampled $T_{1}$ series, regions of interest (ROIs) corresponding to each vial were delineated using the Segment Editor module, generating voxel-by-voxel discretization masks.

Prior to non-linear fitting, the parametric consistency of the signal was verified by analyzing its dependence on the variable acquisition times: pixel intensity variation with TR for R_1_ quantification, and with TE for R_2_ quantification. The fitting functions were implemented in C++ and called from Python via native interfaces. To obtain the R_2_ map, a voxel-by-voxel monoexponential fit was applied according to the equation

(5) *S* = *S*_0_ · *exp*(−*TE* · *R*_2_)

To obtain the R_1_ map, a saturation-recovery fit was applied according to the equation

(6) *S* = *S_0_* · (1 − *exp*(−*TR* · *R*_1_)) · *exp*(−*TE* · *R*_2_)

The parameters were estimated voxel-by-voxel using the Levenberg–Marquardt algorithm, restricting the optimization domain to the segmented region corresponding to the vials. The resulting parametric maps were exported to ImageJ software (version 1.54p) (NIH, Bethesda, MD, USA), where three circular ROIs of 25 × 25 pixels were defined in the central zone of maximum polymerization along the vial axis. To avoid introducing a dose-dependent bias, a fixed geometric criterion (identical in size and relative position across all samples) was established on the control and highest-dose vials and applied uniformly to every sample, though a residual operator-dependent component in the delineation is acknowledged. The placement and geometry of these ROIs guaranteed the statistical representativeness of the signal while minimizing radial polymerization gradients and vial-gel edge artifacts. Finally, the average R_1_ and R_2_ values extracted from the ROIs were linked to the nominal delivered dose to construct the dose–response calibration curves.

The dose–response curves were fitted using linear and second-degree polynomial regressions, reporting the coefficient of determination R^2^ and the sample standard deviation among ROIs for each dose point. The expanded uncertainty is reported as U=k⋅σ with k=2, equivalent to an approximate confidence level of 95% under the assumption of a Gaussian distribution. The R_2_ amplification of Gd-PAGAT relative to PAGAT was calculated as a point-to-point ratio over the common dose points (1 to 10 Gy). The initial sensitivity was estimated as the derivative of the polynomial fit at low dose (D=1 Gy). The choice between the linear and the second-order polynomial model follows from the known dose response of polymer gels rather than from a purely statistical criterion: R_2_ increases in proportion to the radiation-induced polymer fraction, which is linear with dose in the working range and departs from linearity only at higher doses as the monomer is progressively depleted [1,24,26]. The dose sensitivity was therefore taken as the slope of the linear fit in the 1–7 Gy range, reported with its 95% confidence interval, while the second-order polynomial over the full range serves only as an empirical description of the onset of saturation.

### 4.5. Corroborating Spectrophotometric Readout

Following MRI acquisition, the same samples were processed using UV-Vis spectrophotometry as an independent corroboration method. Measurements were performed on a double-beam spectrophotometer using the entire vial as the measurement cuvette, taking advantage of the 4.5 × 1.0 × 1.0 cm^3^ geometry of the transparent polystyrene containers. The measurement wavelength was selected based on the maximum sensitivity of each formulation: 560 nm for conventional PAGAT and 530 nm for Gd-PAGAT. For Gd-PAGAT, this wavelength corresponds to where the optical response of the gel infused with Omniscan proved comparatively more sensitive in preliminary studies.

For PAGAT, three independent replicates were measured per dose point, recording the average of three consecutive spectrophotometric readings for each sample. The reported OD corresponds to the average of the three replicates, and the standard deviation reflects the inter-sample variability. For Gd-PAGAT, due to limited material availability, one sample was measured per dose point with three consecutive readings; in this case, the reported dispersion corresponds to the intra-sample instrumental repeatability. This difference in sampling between formulations is explicitly considered when interpreting the uncertainties associated with the optical readout. The OD–dose curves were fitted using linear and second-degree polynomial regressions in the 1–10 Gy range.

## Figures and Tables

**Table 1:** Average values of the R_2_ map (s^−1^) as a function of dose for conventional PAGAT and Gd-PAGAT at 9 mg/mL, measured with an MSE T_2_ sequence at 1.5 T. Expanded uncertainty at a coverage factor of k = 2, derived from the standard deviation among three ROIs per vial. The value at 0 Gy corresponds to the unirradiated control.

Dose (Gy)	R_2_ PAGAT (s^−1^)	U PAGAT (k = 2)	R_2_ Gd-PAGAT (s^−1^)	U Gd-PAGAT (k = 2)	R_2_ Gd-PAGAT/R_2_ PAGAT
0	0.993	—	52.41	—	2.48 ± 0.42
1	1.280	±0.013	53.18	±0.26	2.89 ± 0.76
3	1.742	±0.091	54.53	±0.61	2.95 ± 0.70
5	2.191	±0.141	55.75	±0.15	2.83 ± 0.68
7	2.430	±0.264	56.32	±0.36	3.04 ± 0.46
10	2.648	±0.309	57.19	±0.08	2.84 ± 0.19

**Table 2:** Optical density as a function of dose for PAGAT (560 nm) and Gd-PAGAT at 9 mg/mL (530 nm), measured by UV-Vis spectrophotometry on the same irradiated samples. Uncertainty is derived from three independent replicates per dose point, each with three instrumental readings.

Dose (Gy)	OD PAGAT (560 nm)	U(OD) PAGAT (k = 2)	OD Gd-PAGAT (530 nm)	U(OD) Gd-PAGAT (k = 2)
1	0.1285	±0.016	0.0045	±0.001
3	0.5535	±0.068	0.4575	±0.002
5	0.8771	±0.138	1.1095	±0.004
7	1.2673	±0.032	1.4360	<0.001
10	1.5365	±0.360	1.6890	<0.001

**Table 3:** Summary of the MRI dosimetric performance of PAGAT and Gd-PAGAT at 9 mg/mL.

Parameter (1.5 T, MSE)	PAGAT	Gd-PAGAT (9 mg/mL)
R_2_ baseline, 0 Gy	0.993 s^−1^	52.41 s^−1^
Dose–R_2_ sensitivity, linear 1–7 Gy	0.1949 s^−1^·Gy^−1^	0.5328 s^−1^·Gy^−1^
Sensitivity amplification (ΔR_2_)	1 (reference)	2.84 ± 0.19
Linear range	1–7 Gy	1–7 Gy
2nd-order polynomial fit (R^2^)	0.9984	0.9969
T_1_ channel (SPGR)	No dose correlation	No dose correlation (T1 saturation)
Optical corroboration (slope difference)	4% (reliable)	Divergent (not reliable)
Practical advantage	Water equivalence, low baseline	Dose-enhancement measurement
Limitation	Lower sensitivity	Higher inter-ROI dispersion

**Table 4:** Acquisition parameters of the quantitative MRI sequences used for the characterization of PAGAT and Gd-PAGAT dosimeters at 1.5 T.

Parameter	MSE (T_2_ Mapping)	SPGR (T_1_ Mapping)
Purpose	R_2_ quantification	R_1_ quantification
TR (ms)	2000	9
TE (ms)	20, 40, 60, 80, 100	4.1
Number of echoes	5	1
Flip angle (°)	90	15 and 30
Acquisition matrix	256 × 181	200 × 200
FOV (mm^2^)	230 × 230	240 × 240
In-plane voxel size (mm^2^)	0.9 × 0.9	0.8 × 0.8
Slice thickness (mm)	5	1
Number of slices	15	65
Acquisition time (min:s)	03:04	03:48

## Data Availability

Data are contained within the article.

## References

[1] De Deene Y. (2022). Radiation Dosimetry by Use of Radiosensitive Hydrogels and Polymers: Mechanisms, State-of-the-Art and Perspective from 3D to 4D. Gels.

[2] Sagsoz M.E., Korkut O., Gallo S. (2025). Advancements in Tissue-Equivalent Gel Dosimeters. Gels.

[3] Venning A.J., Hill B., Brindha S., Healy B.J., Baldock C. (2005). Investigation of the PAGAT Polymer Gel Dosimeter Using Magnetic Resonance Imaging. Phys. Med. Biol..

[4] Deene Y.D. (2009). Review of Quantitative MRI Principles for Gel Dosimetry. J. Phys. Conf. Ser..

[5] Khan M., Heilemann G., Lechner W., Georg D., Berg A.G. (2019). Basic Properties of a New Polymer Gel for 3D-Dosimetry at High Dose-Rates Typical for FFF Irradiation Based on Dithiothreitol and Methacrylic Acid (MAGADIT): Sensitivity, Range, Reproducibility, Accuracy, Dose Rate Effect and Impact of Oxygen Scavenger. Polymers.

[6] Deene Y.D., Mason D. (2023). Optimization of MRI Pulse Sequences and Gadobutrol-Doped Polymer Gel for Real Time 4D Radiation Dosimetry on the MRI-Linac. J. Phys. Conf. Ser..

[7] Senden R.J., De Jean P., McAuley K.B., Schreiner L.J. (2006). Polymer Gel Dosimeters with Reduced Toxicity: A Preliminary Investigation of the NMR and Optical Dose–Response Using Different Monomers. Phys. Med. Biol..

[8] Mesbahi A., Jafarzadeh V., Gharehaghaji N. (2012). Optical and NMR Dose Response of N-Isopropylacrylamide Normoxic Polymer Gel for Radiation Therapy Dosimetry. Rep. Pract. Oncol. Radiother..

[9] Lotfy S., Basfar A.A., Moftah B., Al-Moussa A.A. (2017). Comparative Study of Nuclear Magnetic Resonance and UV–Visible Spectroscopy Dose-Response of Polymer Gel Based on N-(Isobutoxymethyl) Acrylamide. Nucl. Instrum. Methods Phys. Res. Sect. B Beam Interact. Mater. At..

[10] Yoshandi T.M., Razak H.A., Stone N. (2026). A Review Study on Gel Dosimeter Added with Metal-Based Nanoparticles and Its Applications. IOP Conf. Ser. Mater. Sci. Eng..

[11] Zhang D.G., Feygelman V., Moros E.G., Latifi K., Zhang G.G. (2014). Monte Carlo Study of Radiation Dose Enhancement by Gadolinium in Megavoltage and High Dose Rate Radiotherapy. PLoS ONE.

[12] Santibáñez M., Fuentealba M., Vedelago J., Chacón D., Mattea F., Valente M. (2021). Experimental Characterization and Monte Carlo Simulations of the Dose Enhancement on the Millimeter Scale of PAGAT Infused with Gadolinium. Radiat. Phys. Chem..

[13] Taupin F., Flaender M., Delorme R., Brochard T., Mayol J.-F., Arnaud J., Perriat P., Sancey L., Lux F., Barth R.F. (2015). Gadolinium Nanoparticles and Contrast Agent as Radiation Sensitizers. Phys. Med. Biol..

[14] Caravan P., Ellison J.J., McMurry T.J., Lauffer R.B. (1999). Gadolinium(III) Chelates as MRI Contrast Agents: Structure, Dynamics, and Applications. Chem. Rev..

[15] Rogosnitzky M., Branch S. (2016). Gadolinium-Based Contrast Agent Toxicity: A Review of Known and Proposed Mechanisms. BioMetals.

[16] Fuentealba M., Vallejos C., Díez S., Santibáñez M. (2025). Dosimetric Evaluation of the Sensitivity of PAGAT Gel Dosimeters Infused with Clinically Used Gadolinium-Based Contrast Agents. Gels.

[17] Fuentealba M., Ferreira A., Salgado A., Vergara C., Díez S., Santibáñez M. (2024). An Optimized Method for Evaluating the Potential Gd-Nanoparticle Dose Enhancement Produced by Electronic Brachytherapy. Nanomaterials.

[18] Delorme R., Taupin F., Flaender M., Ravanat J.-L., Champion C., Agelou M., Elleaume H. (2017). Comparison of Gadolinium Nanoparticles and Molecular Contrast Agents for Radiation Therapy-Enhancement. Med. Phys..

[19] Sabbaghizadeh R., Shamsudin R., Deyhimihaghighi N., Sedghi A. (2017). Enhancement of Dose Response and Nuclear Magnetic Resonance Image of PAGAT Polymer Gel Dosimeter by Adding Silver Nanoparticles. PLoS ONE.

[20] Mustaqim A.S., Yahaya N.Z., Razak N.N.A., Zin H. (2020). The Dose Enhancement of MAGAT Gel Dosimeter Doped with Zinc Oxide at 6 MV Photon Beam. Radiat. Phys. Chem..

[21] Rand S., Maravilla K.R., Schmiedl U. (1994). Lesion enhancement in radio-frequency spoiled gradient-echo imaging: Theory, experimental evaluation, and clinical implications. Am. J. Neuroradiol..

[22] Hathout G., Jamshidi N. (2012). Parameter Optimization for Quantitative Signal-Concentration Mapping Using Spoiled Gradient Echo MRI. Radiol. Res. Pract..

[23] Marth T., Froehlich J.M., Nanz D., Sutter R. (2025). Gadolinium Concentration Dependent Signal Enhancement Profiles Using Routine Clinical Sequences with Gadopiclenol, Gadoterate, Gadobutrol, and Gadoxetate at 1.5, 3 and 7 Tesla. Eur. J. Radiol..

[24] Macchione M.A., Lechón Páez S., Strumia M.C., Valente M., Mattea F. (2022). Chemical Overview of Gel Dosimetry Systems: A Comprehensive Review. Gels.

[25] Maryañski M.J., Zastavker Y.Z., Gore J.C. (1996). Radiation Dose Distributions in Three Dimensions from Tomographic Optical Density Scanning of Polymer Gels: II. Optical Properties of the BANG Polymer Gel. Phys. Med. Biol..

[26] Baldock C., De Deene Y., Doran S., Ibbott G., Jirasek A., Lepage M., McAuley K.B., Oldham M., Schreiner L.J. (2010). Polymer Gel Dosimetry. Phys. Med. Biol..

[27] Baldock C., Lepage M., Bäck S.Å.J., Murry P.J., Jayasekera P.M., Porter D., Kron T. (2001). Dose resolution in radiotherapy polymer gel dosimetry: Effect of echo spacing in MRI pulse sequence. Phys. Med. Biol..

[28] Deene Y.D., Walle R.V.D., Achten E., Wagter C.D. (1998). Mathematical Analysis and Experimental Investigation of Noise in Quantitative Magnetic Resonance Imaging Applied in Polymer Gel Dosimetry. Signal Process..

[29] Venning A.J., Brindha S., Hill B., Baldock C. (2004). Preliminary Study of a Normoxic PAG Gel Dosimeter with Tetrakis (Hydroxymethyl) Phosphonium Chloride as an Anti-Oxidant. J. Phys. Conf. Ser..

[30] IAEA (2024). Absorbed Dose Determination in External Beam Radiotherapy.

